# Clinical factors and dopamine transporter availability for the prediction of outcomes after globus pallidus deep brain stimulation in Parkinson’s disease

**DOI:** 10.1038/s41598-022-19150-3

**Published:** 2022-10-07

**Authors:** Seung Hyun Lee, Mina Kim, Jooyoung Lee, Jae-Woo Kim, Mi Sun Kim, Sungyang Jo, Sang Ryong Jeon, Sun Ju Chung

**Affiliations:** 1 Department of Neurology, Seoul Medical Center, Seoul, Korea; 2 Department of Applied Statistics, Chung-Ang University, Seoul, Korea; 3 Department of Neurology, Asan Medical Center, University of Ulsan College of Medicine, Seoul, Korea; 4 Department of Neurological Surgery, Asan Medical Center, University of Ulsan College of Medicine, Seoul, Korea

**Keywords:** Neurology, Neurological disorders

## Abstract

We aimed to investigate the predictive value of preoperative clinical factors and dopamine transporter imaging for outcomes after globus pallidus interna (GPi) deep brain stimulation (DBS) in patients with advanced Parkinson’s disease (PD). Thirty-one patients with PD who received bilateral GPi DBS were included. The patients underwent preoperative [^18^F] FP-CIT positron emission tomography before DBS surgery. The Unified Parkinson’s Disease Rating Scale (UPDRS) were used to assess outcomes 12 months after DBS. Univariate and multivariate linear regression analysis were performed to investigate the association between clinical variables including sex, age at onset of PD, disease duration, cognitive status, preoperative motor severity, levodopa responsiveness, daily dose of dopaminergic medication, and dopamine transporter availability in the striatum and outcomes after GPi DBS. Younger age at onset of PD was associated with greater DBS motor responsiveness and lower postoperative UPDRS III score. Greater levodopa responsiveness, lower preoperative UPDRS III score and lower striatal dopamine transporter availability were associated with lower postoperative UPDRS III score. Younger age at onset was also associated with greater decrease in UPDRS IV score and dyskinesia score after GPi DBS. Our results provide useful information to select DBS candidates and predict therapeutic outcomes after GPi DBS in advanced PD.

## Introduction

Deep brain stimulation (DBS) is an established surgical treatment for patients with advanced Parkinson’s disease (PD) complicated with symptoms of motor fluctuation or levodopa-induced dyskinesia (LID) not adequately controlled with optimal medication^1,2^. The subthalamic nucleus (STN) and globus pallidus interna (GPi) are most studied targets for DBS in PD, and no difference between stimulation of the two targets in improvement in motor symptoms was found^2^. Preoperative levodopa therapeutic responsiveness, which are largely correlated with the extent of improvement in motor symptoms after DBS surgery, is a critical component of DBS candidate selection and predicting outcomes after DBS^2–5^. Other clinical factors, including age at surgery, preoperative Unified Parkinson’s Disease Rating Scale (UPDRS) motor scores in the medication off state, disease duration, and phenotype of PD have been suggested as predictors of outcomes after DBS^6–8^. However, since most of the studies conducted on patients who underwent STN DBS^6,7,9^, the predictive factors for outcomes after GPi DBS have not been adequately studied. Moreover, the factors associated with the postoperative changes in motor complications after DBS remain unclear.

The pathological hallmark of PD is the loss of dopaminergic neurons in the midbrain and the presence of Lewy bodies in the cytoplasm of the surviving neurons^10^. Functional neuroimaging can be used to assess the integrity of dopamine neurons in the midbrain, such as dopamine transporter imaging using single-photon emission computed tomography (SPECT) or positron emission tomography (PET) scans^11–14^. As a prognostic imaging biomarker, dopamine transporter imaging is associated with disease progression and long-term outcomes of PD^15–17^. Furthermore, previous studies found that presynaptic dopaminergic depletion is associated with an increased risk for later motor complication in PD^18,19^. However, despite its clinical utility, the role of dopamine transporter imaging in predicting outcomes after DBS have not been established yet^4,5^.

Therefore, the aim of the current study is to investigate the predictive values of preoperative clinical factors and dopamine transporter imaging for outcomes after DBS in patients with advanced PD who underwent GPi DBS.

## Materials and methods

### Study design

We retrospectively reviewed 76 patients with advanced PD who underwent bilateral GPi DBS between January 2013 and December 2019 at the Asan Medical Center, Seoul, Korea. The inclusion criteria were (1) the diagnosis of PD was based on the UK Brain Bank criteria, (2) no prior history of brain surgery, (3) preoperative evaluation including brain magnetic resonance imaging (MRI) and N-(3-[18F] fluoropropyl)-2β-carbon ethoxy-3β-(4-iodophenyl) nortropane ([^18^F] FP-CIT). Among the 76 study participants, we excluded 15 patients who were unable to use pre-contrast three-dimensional T1 imaging data, and 14 patients had [^18^F] FP-CIT PET performed at another institution, and 16 patients who underwent [^18^F] FP-CIT PET before preoperative evaluation from this study. Therefore, 31 patients (16 women, 15 men) were included in the final analysis (Supplementary Fig. 1). They were also classified into two sub-groups, young-onset PD and older-onset PD, according to age at onset of PD based on the age of 50^20^.

### Perioperative procedures

All patients were admitted 3–6 months before DBS surgery for preoperative evaluation. A standardized oral levodopa challenge test was conducted. Evaluation of medication off state was then conducted after more than 12 h of deprivation of dopaminergic medication. After evaluation of the medication off state, 1.5-fold the usual first morning levodopa equivalent dose (LED) was administered to evaluate the medication on state. The selection of target sites (GPi vs STN) was determined by an individual risk–benefit evaluation for each patient and a consensus decision made by physicians, patients, and their caregivers. In general, GPi was not preferred for patients who needed dopaminergic medication reduction or had severe psychiatric comorbidities related to dopaminergic medication. In addition, patients with severe cognitive dysfunction or uncontrolled psychiatric disease were not subject to DBS in our center. All patients underwent DBS according to the institutional guidelines for this surgery. Stereotactic implantation of bilateral GPi DBS (Activa 3387, Medtronic, Minneapolis, MN, USA) was performed under local anesthesia with microelectrode recording (MER) guidance. The patients underwent initial DBS programming three weeks after surgery and was followed to optimize stimulation parameters and medication by visits every three to four months or if clinically necessary.

### Image acquisition and quantification

All subjects underwent [^18^F] FP-CIT PET (Biograph TruePoint 40, Siemens Medical System) as described previously^21^. Quantitative image processing was then conducted using Statistical Parametric Mapping (SPM12, Wellcome Department of Imaging Neuroscience, Institute of Neurology, London, UK) in MATLAB (MathWorks, Inc. Natick, MA) software. PET scan images were co-registered with each patient’s structural brain MRI. All reconstructed PET scan images were normalized to the template in the Montreal Neurological Institute (MNI) standard space. The specific uptake of [^18^F] FP-CIT into the striatal regions was also calculated using region of interest (ROI) analysis. Mean counts per voxel were extracted for each ROI based on the automated anatomical labeling atlas of structural ROIs using the MarsBaR toolbox (http://marsbar.sourceforge.net)^22^. Subsequently, the dopamine transporter availability in the caudate and putamen of each brain hemisphere was calculated in terms of the specific binding ratio (SBR) as follows: Dopamine transporter availability = (mean counts of caudate ROI or putamen ROI–mean counts of occipital ROI)/(mean counts of occipital ROI). The occipital cortex was used as a reference region^23^. The sum of SBR values of putamen and caudate was averaged to calculate mean SBR value in the striatum.

### Outcome measures

12 months after GPi DBS, patients were assessed UPDRS III in four conditions to: (1) medication off stimulation off, (2) medication off stimulation on, (3) medication on stimulation off and (4) medication on stimulation on state. Switching of stimulation was performed at interval of about 30 min. The primary outcome of this study was preoperative factors associated with postoperative DBS motor responsiveness 12 months after surgery. The postoperative DBS motor responsiveness was defined as the percentage change between the UPDRS III scores in the medication off stimulation on state 12 months after GPi DBS and the preoperative UPDRS III scores in the medication off state. The secondary outcomes of this study were preoperative factors associated with postoperative UPDRS III scores and IV score 12 months after DBS surgery. The UPDRS III scores in the medication on stimulation on state, and in the medication off stimulation on state were selected for measuring motor outcomes after GPi DBS. Changes in the UPDRS IV scores were used to compare dyskinesia (item 32–35) and motor fluctuation (item 36–39). LEDD was calculated according to the previously established conversion formula^24^.

### Statistical analysis

Baseline demographic and clinical data were presented as mean ± standard deviation. We confirmed the normal distribution of variables using the Shapiro–Wilk test and variable histograms. Baseline and postoperative clinical scores were compared using paired t-tests and or Wilcoxon signed-rank tests. Correlation between variables was analyzed using Pearson’s correlation coefficient. Comparison between the young-onset PD group and the older-onset PD group was performed using the student t-test or Mann–Whitney test. The Benjamini–Hochberg procedure to control false positive rate (FDR) at 0.05 was used for multiple comparison correction. We conducted univariate and multivariate linear regression analysis to investigate the association between preoperative variables, including sex, age at onset of PD, disease duration, MMSE score, preoperative UPDRS III score in the medication off state, levodopa responsiveness, LEDD, and the mean SBR value in the striatum and outcomes after GPi DBS. All data were analyzed using SPSS version 22.0 (version 22.0; IBM Corp., Armonk, NY, USA) and R (4.1.0). The significance level was set at p < 0.05.

### Ethical approval and informed consent

All procedures performed in the study involving human participants was accordance with the ethical standards of the institutional and national research committee. The study was performed in accordance with the 1964 Helsinki Declaration and its later amendments or comparable ethical standards. The requirement for patient consent was waived due to the retrospective nature of the study.

### Institutional approval

The Institutional Review Board (IRB) of the Asan Medical Center approved this study (IRB No. 2021–0017).

## Results

### Clinical characteristics, striatal SBR value and outcomes after GPi DBS

The clinical characteristics and DBS outcomes of the 31 patients with PD included in this study are summarized in Table 1. The mean age at onset of PD was 48.52 ± 6.88 years and the mean age at GPi DBS surgery was 62.45 ± 6.13 years. The disease duration from symptom onset to DBS was 13.94 ± 4.29 years. The mean score of MMSE was 26.61 ± 3.24. The mean preoperative levodopa responsiveness was 64.78 ± 15.54%. The mean SBR value in the striatum was 0.82 ± 0.40.

**Table 1:** Baseline characteristics and outcomes after GPi DBS in patients with advanced Parkinson’s disease.

PD patients (n = 31)	Baseline	12 months after GPi DBS	p	adjusted p
Women (%)	16 (51.62)			
Age at onset (years)	48.52 ± 6.88			
Disease duration (years)	13.94 ± 4.29			
Age at surgery (years)	62.45 ± 6.13			
Mini-Mental State Examination (MMSE)	26.61 ± 3.24			
Levodopa responsiveness (%)	64.78 ± 15.54			
Hoehn & Yahr stage (medication off)	3.52 ± 0.53			
Striatal SBR value	0.82 ± 0.40			
UPDRS III	Medication off	Medication off stimulation on		
	44.95 ± 13.35	31.69 ± 10.99	< 0.001	< 0.001
		Medication off stimulation off		
		40.06 ± 12.05	0.060	0.080
UPDRS III	Medication on	Medication on stimulation on		
	16.45 ± 1.68	17.66 ± 10.26	0.108	0.123
		Medication on stimulation off		
		23.50 ± 11.62	< 0.001	< 0.001
DBS motor responsiveness (%)		26.71 ± 25.27		
UPDRS IV	8.58 ± 2.38	4.94 ± 1.91	< 0.001	< 0.001
Dyskinesia	2.87 ± 1.98	1.32 ± 1.25	< 0.001	< 0.001
Motor fluctuation	4.58 ± 1.06	2.58 ± 1.39	< 0.001	< 0.001
LEDD (mg)	1199.61 ± 265.95	1194.61 ± 333.08	0.919	0.919

Values are reported as mean ± standard deviation or number of patients (%). DBS, Deep brain stimulation; GPi, Globus pallidus interna; LEDD, Levodopa equivalent daily dose; MMSE, Mini-Mental State Examination; PD, Parkinson’s disease; SBR, Specific binding ratio; UPDRS, Unified Parkinson’s Disease Rating Scale.

12 months after surgery, GPi DBS significantly improved UPDRS III score in the medication off state (44.95 ± 13.35 vs 31.69 ± 10.99, p < 0.001). There was no statistical difference between the preoperative UPDRS III score in the medication off state and the postoperative UPDRS III score in the medication off stimulation off state (44.95 ± 13.35 vs 40.06 ± 12.05, p = 0.060). In addition, there was no difference between the preoperative UPDRS III score in the medication on state and postoperative UPDRS III score in the medication on stimulation on state (16.45 ± 1.68 vs 17.66 ± 10.26, p = 0.108). However, the UPDRS III score in the medication on stimulation off state 12 months after surgery showed a significant increase compared with preoperative medication on state (16.45 ± 1.68 vs 23.50 ± 11.62, p < 0.001). Mean postoperative DBS responsiveness was 26.71 ± 25.71%. Lastly, GPi DBS significantly improved UPDRS IV scores compared with baseline, and dyskinesia and motor fluctuation scores were also improved. Additional information on the motor phenotypes and DBS programming parameters of the PD patients included in this study is in Supplementary table 1.

### Preoperative predictors of motor responsiveness after GPi DBS

**Figure 1:**
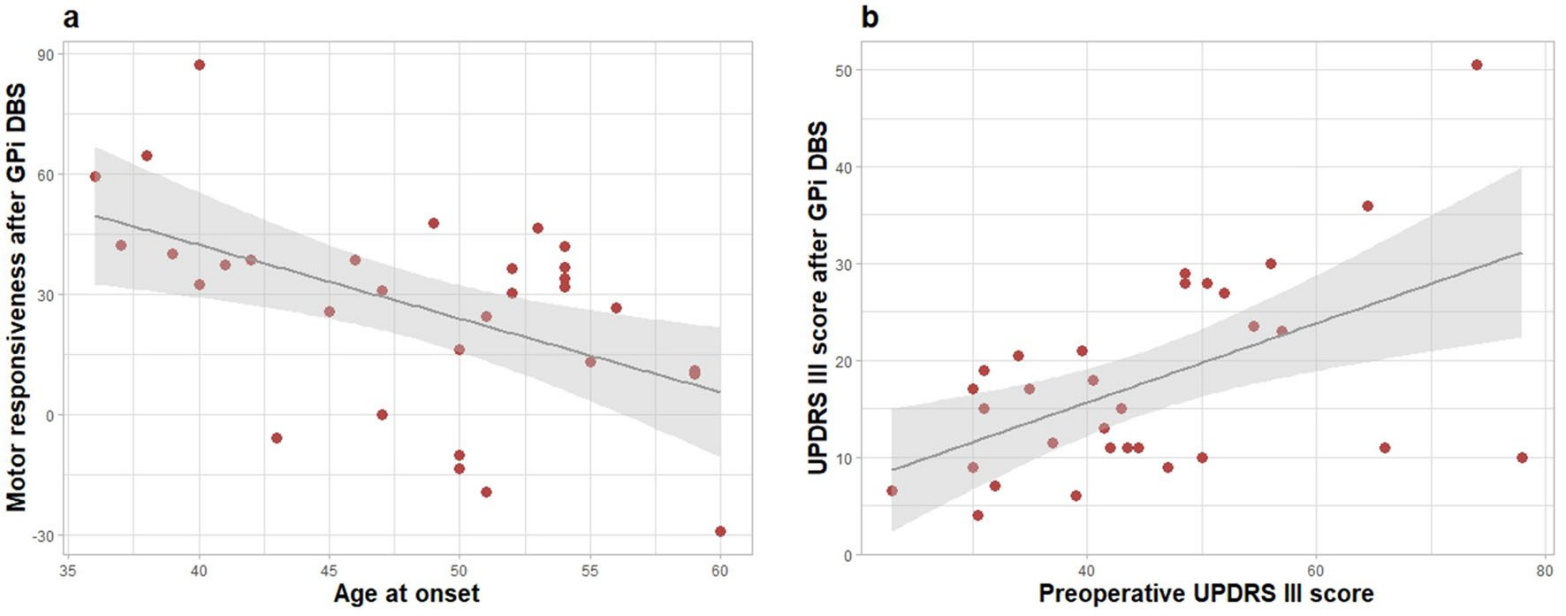
(**a**) Scatter plot of age at onset of PD and motor responsiveness 12 months after GPi DBS. There is a negative linear relationship beteween these two variables. Pearson’s r = −0.50, p = 0.004. (**b**) Scatter plot of preoperative UPDRS III in the medication off state and postoperative UPDRS III in the medication on stimulation on state 12 months after GPi DBS. There is a positive linear relationship between these two variables. Pearson’s r = 0.53, p = 0.002. The correlation is significant at a 0.05 level. PD = Parkinson’s disease, GPi = Globus pallidus interna, DBS = Deep brain stimulation, UPDRS = Unified Parkinson’s Disease Rating Scale.

Younger age at onset of PD was associated with greater DBS motor responsiveness in both univariate and multivariate analyses, and higher preoperative UPDRS III in the medication off state was associated with better DBS motor responsiveness in the univariate analysis only (Table 2). Figure 1a shows a negative linear relationship between age at onset of PD and motor responsiveness after GPi DBS (Pearson’s r = −0.50, p = 0.004).

**Table 2:** Univariable and multivariate regression analysis of the factors associated with the DBS motor responsiveness in patients with advanced Parkinson’s disease who underwent GPi DBS.

	Univariate analysis			Multivariate analysis		
	Coefficient	95% CI	p	Coefficient	95% CI	p
Sex (male)	17.621	(−0.05, 35.30)	0.051	14.417	(−4.39, 33.22)	0.126
Age at onset	−1.837	(−3.04, −0.63)	0.004	−1.712	(−3.13, −0.29)	0.020
Disease duration	2.072	(−0.02, 4.17)	0.052	0.385	(−2.45, 3.22)	0.781
MMSE	−0.84	(−3.78, 2.10)	0.564	−1.586	(−4.58, 1.41)	0.283
Preoperative UPDRS III score	0.726	(0.06, 1.39)	0.033	0.419	(−0.36, 1.20)	0.278
Levodopa responsiveness (%)	−0.053	(−0.67, 0.56)	0.861	0.185	(−0.59, 0.96)	0.624
LEDD (mg)	0.011	(−0.03, 0.05)	0.553	−0.0002	(−0.04, 0.04)	0.993
Striatal SBR value	−3.669	(−27.46, 20.13)	0.75	3.846	(−24.01, 31.70)	0.78

DBS, deep brain stimulation; GPi, globus pallidus interna; MMSE, Mini-Mental State Examination; SBR, specific binding ratio; LEDD, levodopa equivalent daily dose; UPDRS, Unified Parkinson’s Disease Rating Scale.

### Preoperative predictors of UPDRS III scores after GPi DBS

In the medication on stimulation on state 12 months after GPi DBS, lower preoperative UPDRS III score in the medication off state and greater levodopa responsiveness were associated with lower UPDRS III score in both univariate and multivariate analyses (Table 3). Younger age at onset of PD and lower striatal SBR value were associated with lower UPDRS III score in multivariate analysis only. Higher MMSE score was associated with lower UPDRS III in univariate analysis, but not in multivariate analysis. Figure 1b shows a positive linear relationship between preoperative UPDRS III score in the medication off state and postoperative UPDRS III score in the medication on stimulation on state (Pearson’s r = 0.53, p = 0.002). Scatter plot for levodopa responsiveness and postoperative UPDRS III score in the medication on stimulation on state is in Supplementary Fig. 2.

**Table 3:** Univariable and multivariate regression analysis of the factors associated with the UPDRS III scores in the medication on stimulation on state 12 months after GPi DBS in patients with advanced Parkinson' disease.

	Univariate analysis			Multivariate analysis		
	Coefficient	95% CI	p	Coefficient	95% CI	p
Sex (male)	−4.51	(−11.99, 2.97)	0.227	−1.529	(−7.48, 4.42)	0.599
Age at onset	0.39	(−0.16, 0.94)	0.155	0.624	(0.18, 1.07)	0.009
Disease duration	0.319	(−0.58, 1.22)	0.475	0.453	(−0.44, 1.35)	0.306
MMSE	−1.429	(−2.50, −0.36)	0.010	−0.926	(−1.87, 0.02)	0.055
Preoperative UPDRS III score	0.409	(0.16, 0.66)	0.002	0.279	(0.03, 0.53)	0.029
Levodopa responsiveness (%)	−0.275	(−0.50, −0.05)	0.02	−0.272	(−0.52, −0.03)	0.031
LEDD (mg)	−0.009	(−0.02, 0.01)	0.191	−0.003	(−0.02, 0.01)	0.605
Striatal SBR value	0.364	(−9.32, 10.05)	0.939	9.013	(0.20, 17.82)	0.045

DBS, deep brain stimulation; GPi, globus pallidus interna; MMSE, Mini-Mental State Examination; SBR, specific binding ratio; LEDD, levodopa equivalent daily dose; UPDRS, Unified Parkinson’s Disease Rating Scale.

In the medication off stimulation on state 12 months after GPi DBS, younger age at onset of PD was associated with lower UPDRS III score in multivariate analysis only (supplementary table 1). Lower preoperative UPDRS III score in the medication off state was associated with lower UPDRS III score in both univariate and multivariate analyses.

### Preoperative predictors of changes in UPDRS IV scores after GPi DBS

Younger age at onset of PD was associated with greater decrease in UPDRS IV scores 12 months after GPi DBS in both univariate and multivariate analyses (Table 4). There is a negative linear relationship between the age at onset of PD and changes in UPDRS IV score after GPi DBS (Supplementary Fig. 3, Pearson’s r = −0.39, p = 0.032). Furthermore, younger age at onset of PD was associated with greater decrease in dyskinesia scores in multivariate analysis (Supplementary table 2). However, no significant variable was associated with changes in motor fluctuation scores in both univariate and multivariate analyses.

**Table 4:** Univariable and multivariate regression analysis of the factors associated with the changes in UPDRS IV scores 12 months after GPi DBS in patients with advanced Parkinson' disease.

	Univariate analysis			Multivariate analysis		
	Coefficient	95% CI	p	Coefficient	95% CI	p
Sex (male)	1.075	(−0.76, 2.91)	0.240	0.41	(−1.60, 2.42)	0.676
Age at onset	−0.141	(−0.27, −0.01)	0.032	−0.203	(−0.36, −0.05)	0.011
Disease duration	0.011	(−0.21, 0.23)	0.917	−0.194	(−0.50, 0.11)	0.197
MMSE	0.018	(−0.28, 0.31)	0.900	0.002	(−0.32, 0.32)	0.991
Preoperative UPDRS III score	0.012	(−0.06, 0.08)	0.728	0.041	(−0.04, 0.12)	0.320
Levodopa responsiveness (%)	0.025	(−0.04, 0.09)	0.411	0.08	(0.00, −0.16)	0.058
LEDD (mg)	−0.0003	(0.00, 0.00)	0.875	−0.002	(−0.01, 0.00)	0.422
Striatal SBR value	−0.818	(−3.17, 1.53)	0.482	−2.458	(−5.43, 0.52)	0.101

DBS, deep brain stimulation; GPi, globus pallidus interna; MMSE, Mini-Mental State Examination; SBR, specific binding ratio; LEDD, levodopa equivalent daily dose; UPDRS, Unified Parkinson’s Disease Rating Scale.

### Comparison between young-onset PD and older-onset PD

We compared young-onset PD patients and older-onset PD patients, and the results are summarized in Supplementary table 4. 14 young-onset PD patients and 17 older-onset PD patients were identified in our cohort, age at onset of PD was 42.14 ± 4.11 years in the young-onset PD group and 53.76 ± 3.21 in the older-onset PD group (p < 0.001). There were no significant differences between the two groups in preoperative UPDRS III and IV scores. Striatal dopamine transporter availability was 0.68 ± 0.35 in young-onset PD group and 0.94 ± 0.42 in older-onset PD group, but there was no statistical difference between the two groups (p = 0.080). The UPDRS III score in the medication off stimulation off state 12 months after GPi DBS was significantly lower in the young-onset PD group compared with the older-onset PD group (35.35 ± 12.41 vs 43.94 ± 10.57, p = 0.046), but it was not significant after correction for multiple comparisons (adjusted p = 0.212). There was no difference between the two groups in postoperative UPDRES III scores in other conditions. GPi motor responsiveness was higher in the young-onset PD group compared to the older-onset PD group (38.54 ± 23.69% vs 16.97 ± 22.77%, p = 0.015), however, after correction it was not significant (adjusted p = 0.086). There was no difference in UPDRS IV score between the groups 12 months after surgery.

## Discussion

The present study demonstrated that age at onset of PD was associated with motor outcomes of GPi DBS 12 months after surgery in patients with advanced PD. The levodopa responsiveness, UPDRS III scores in the medication off state, and striatal dopamine transporter availability were also preoperative predictors of motor outcomes after GPi DBS. In addition, age at onset of PD was also associated with the postoperative changes in UPDRS IV scores and dyskinesia scores.

Evidence suggests that the best outcomes after DBS are associated with excellent levodopa responsiveness, younger age, no or few axial motor symptoms, no cognitive impairment, and absence of or well-controlled psychiatric disease at baseline in patients with advanced PD^3–5^. However, most previous studies enrolled patients who underwent STN DBS, therefore, predictive value of clinical factors associated with the outcome of GPi DBS in patients with PD remain unclear. In one study with 20 PD patients who underwent GPi DBS, preoperative levodopa responsiveness correlated with response to DBS on the Movement Disorder society (MDS) UPDRS III scores in the medication off state, but not on the non-tremor total scores 12 months after surgery^25^. Another study with 65 PD patients reported that GPi DBS improved motor function regardless of motor subtypes, but the improvement was greater in the patients with tremor dominant (TD) phenotype at 12 months after surgery^26^. However, those studies have limitations because they did not correct for variables that can affect the outcomes after DBS, such as disease duration, preoperative motor severity as well as levodopa responsiveness^6,7^. In our multivariate analysis, younger age at onset of PD was a consistent predictor of better motor outcomes in both postoperative DBS motor responsiveness and UPDRS III scores. Lower preoperative UPDRS III score in the medication off state was associated with the lower postoperative UPDRS III scores. The greater levodopa responsiveness was a predictive factor of lower postoperative UPDRS III scores in the medication on stimulation on state, but there was no association between levodopa responsiveness and postoperative DBS motor responsiveness or UPDRS III scores in the medication off stimulation on state. Similarly, the striatal SBR value was only associated with the UPDRS III score in the medication on stimulation on state 12 months after surgery. Higher preoperative UPDRS III score in the medication off state was associated with better GPi DBS motor responsiveness in univariate analysis, but not in multivariate analysis. Also, higher MMSE score was associated with lower postoperative UPDRS III score in the medication on stimulation on state only in univariate analysis. Male sex, disease duration, and LEDD did not contribute to prediction of motor outcome after GPi DBS in our cohort.

Although dopamine transporter imaging has been suggested to have a prognostic value in PD, the role of dopamine transporter imaging in the selection of candidates for DBS and the prediction of the outcomes of DBS has not been well established. Nakajima et al. studied dopamine transporter imaging in patients PD who underwent STN DBS^27^. From their results, no correlation was identified between the SBR value of the striatum and UPDRS III score in the medication on stimulation on state after DBS surgery. In this study, the striatal SBR value was not related to the motor outcomes after GPi DBS in univariate analysis. However, the striatal SBR value identified as a predictor of the postoperative UPDRS III score after other clinical variables were adjusted. Interestingly, lower striatal SBR value was associated with better UPDRS III scores after GPi DBS (Table 3, coefficient 9.013. p = 0.045) in our study. In a previous PET imaging study using presynaptic dopamine markers, researchers reported that the loss of putamen [^11^C] ( ±) dihydrotetrabenazine (DTBZ) binding at symptom onset was substantially greater in patients with younger-onset PD, however, the rate of DTBZ binding loss was lower than in patients with older-onset PD^28^. Palermo et al. reported that patients with younger-onset PD exhibited more reduced dopamine transporter binding in the putamen than those with older-onset PD. However, these presentations were not related to the severity of motor symptoms, suggesting age-related differences in striatal compensatory mechanisms in PD^29^. There is evidence that the loss of dopaminergic neurons is functionally compensated for by downregulation of the dopamine transporter, which reduces dopamine reuptake into presynaptic dopamine nerve terminals^30^. Young-onset PD patients are presumed to have more efficient compensatory ability than older-onset PD patients^27,28^, and our results may also be related to age-specific heterogeneity in compensatory mechanisms for dopaminergic denervation. However, in our cohort, there was no statistical difference in the striatal SBR value or postoperative UPDRS III scores between the young-onset PD group and older-onset PD group. This result may be attributable to the small number of patients in our cohort. Further studies are needed to determine the effect of age at onset of PD on the relationship between striatal dopamine transporter availability and motor outcome after DBS. Furthermore, among the significant predictors, the striatal SBR value showed a weak association with the postoperative UPDRS III score in the medication on stimulation on state in multivariate analysis. These results suggest that the clinical usefulness of preoperative dopamine transporter imaging for predicting motor outcome after DBS may be limited compared with clinical predictors such as age at onset of PD, levodopa responsiveness, and preoperative UPDRS III score.

In case of PD, DBS targets patients with advanced stage who presented with motor complications that are not well controlled by medication. Previous studies demonstrated that DBS improves not only motor symptoms but also increases the duration of “on” time^31,32^. However, the clinical predictors associated with changes in motor fluctuation and dyskinesia after DBS are not clear. Especially, although GPi DBS has a direct dyskinesia suppression effect^33^, preoperative factors associated with the effect of GPi DBS on dyskinesia after surgery have not been well studied. In our study, younger age at onset of PD was associated with greater decrease in UPDRS IV total score and dyskinesia score 12 months after GPi DBS. Young-onset PD patients may have milder motor symptoms, but earlier appearance of motor complications compared with older-onset PD patients^28,29^. Nevertheless, our data suggest that GPi stimulation can effectively ameliorated motor complications in younger onset PD patients, especially on dyskinesia. On the other hand, there was no difference in LEDD at baseline and 12 months after DBS surgery in this study, which was consistent with previous results^25^. This provides additional evidence that GPi DBS has an advantage for flexibility in medication adjustments with direct anti-dyskinetic effect^2^.

Our study has several limitations. First, this study was retrospectively analyzed, using a limited number of patients. In particular, eight variables were included in the multiple regression analysis of this study, but the relatively small number of subjects limits the statistical power of the multiple regression model. Second, the age at onset of PD of our cohort was relatively young at 48.5 years, and 14 out of 31 patients with young-onset PD patients were included. Therefore, caution is needed in generalizing our results for older-onset PD patients. Third, although all patients in this study underwent dopamine transporter imaging 3 to 6 months before DBS surgery, however, the duration from symptom onset of PD to dopamine transporter imaging varied. Therefore, further larger-scale and controlled studies are needed to establish the association of presynaptic dopaminergic depletion and motor outcomes of DBS. Fourth, although we adjusted for several clinical variables and striatal dopamine transporter availability in the multivariate linear regression analysis, there were still other possible confounders that can affect the outcomes of DBS, including genotypes of PD, motor phenotypes of PD, postoperative lead location and DBS programming parameters that were not considered in the analysis of our study. Fifth, since UPDRS IV score is measured by the patient’s report, it is necessary to verify our results by more objective measurement of motor complications in the future studies.

## Conclusion

Our study suggests that age at onset of PD, preoperative motor severity, levodopa responsiveness and striatal dopamine transporter availability at baseline have predictive value for outcomes after GPi DBS in patients with advanced PD. These results provide additional evidence that the outcomes after DBS may vary depending on the clinical heterogeneity of PD.

## Supplementary Information

Supplementary Information.

## Data Availability

The datasets generated during and analyzed during the current study are available from the corresponding author on resonable request.
