# Supplementary material for: Clinical factors and dopamine transporter availability for the prediction of outcomes after globus pallidus deep brain stimulation in Parkinson’s disease
Source: Sci Rep. 2022 Oct 7;12:16870. doi: 10.1038/s41598-022-19150-3 (PMC9547008; doi:10.1038/s41598-022-19150-3)
Supplement: Supplementary file 1 — Supplementary Information. [file 41598_2022_19150_MOESM1_ESM.docx]

**Supplementary Figures and Tables**

**Table of contents**

| Supplementary Figure 1 | The study flow chart. |
| --- | --- |
| Supplementary Figure 2 | The correlation between levodopa responsiveness and UPDRS III score in the medication on stimulation on state after GPi DBS in patients with advanced PD |
| Supplementary Figure 3 | The correlation between age at onset of PD and changes in UPDRS IV score in the after GPi DBS in patients with advanced PD. |
| Supplementary Table 1 | Clinical features, motor phenotypes and DBS programming parameters at 12 months after surgery in PD patients who underwent GPi DBS. |
| Supplementary Table 2 | Univariable and multivariate regression analysis of the factors associated with the UPDRS III scores in the medication off stimulation on state 12 months after GPi DBS in patients with advanced Parkinson' disease. |
| Supplementary Table 3 | Univariable and multivariate regression analysis of the factors associated with the changes in dyskinesia scores 12 months after GPi DBS in patients with advanced Parkinson' disease. |
| Supplementary Table 4 | Univariable and multivariate regression analysis of the factors associated with the changes in motor fluctuation scores 12 months after GPi DBS in patients with advanced Parkinson' disease. |
| Supplementary Table 5 | Comparison between younger-onset PD patients and older-onset PD patients who underwent GPi DBS |


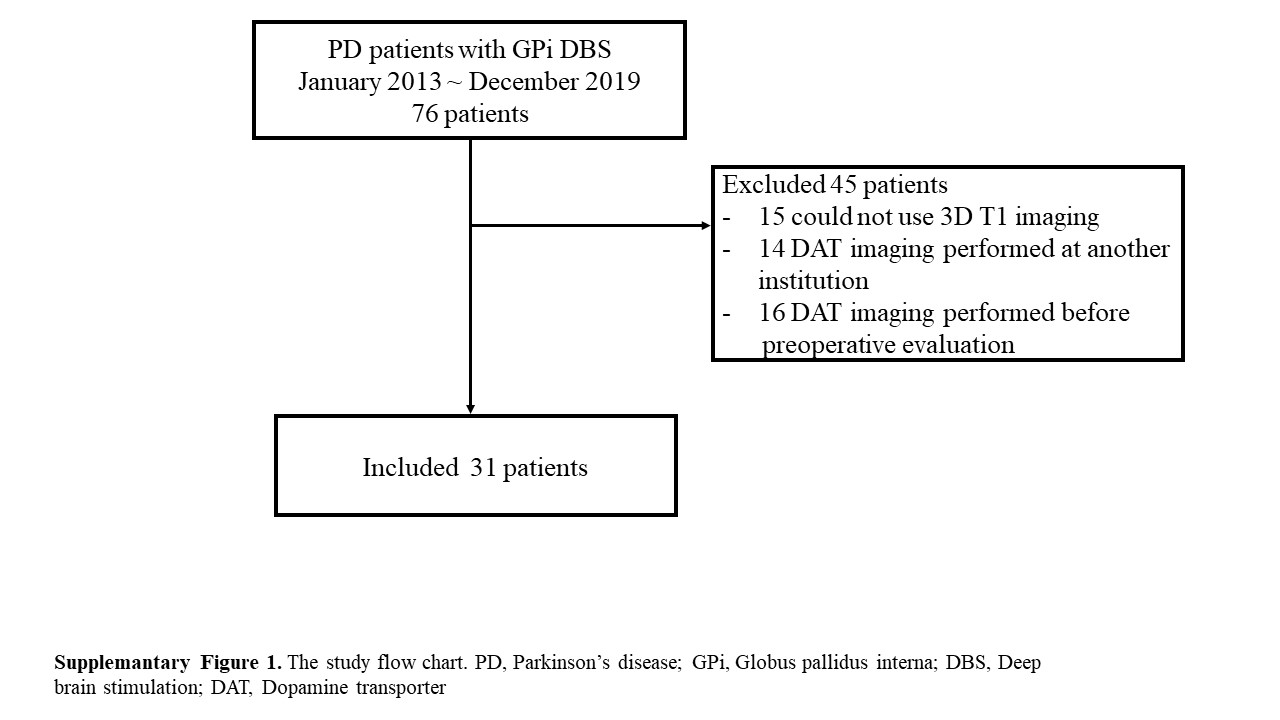


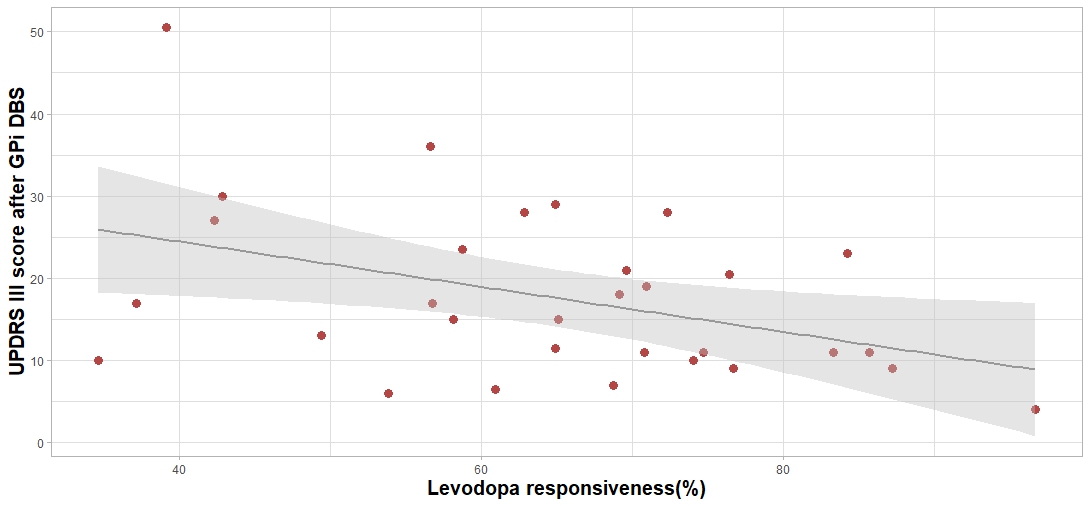


**Supplementary Figure 2.** The correlation between levodopa responsiveness and UPDRS III score in the medication on stimulation on state after GPi DBS in patients with advanced PD. There is a negative linear relationship between two variables. Pearson’s r = -0.42, p = 0.02. The correlation is significant at a 0.05 level. PD = Parkinson’s disease, GPi = Globus pallidus interna, DBS = Deep brain stimulation, UPDRS = Unified Parkinson’s Disease Rating Scale.


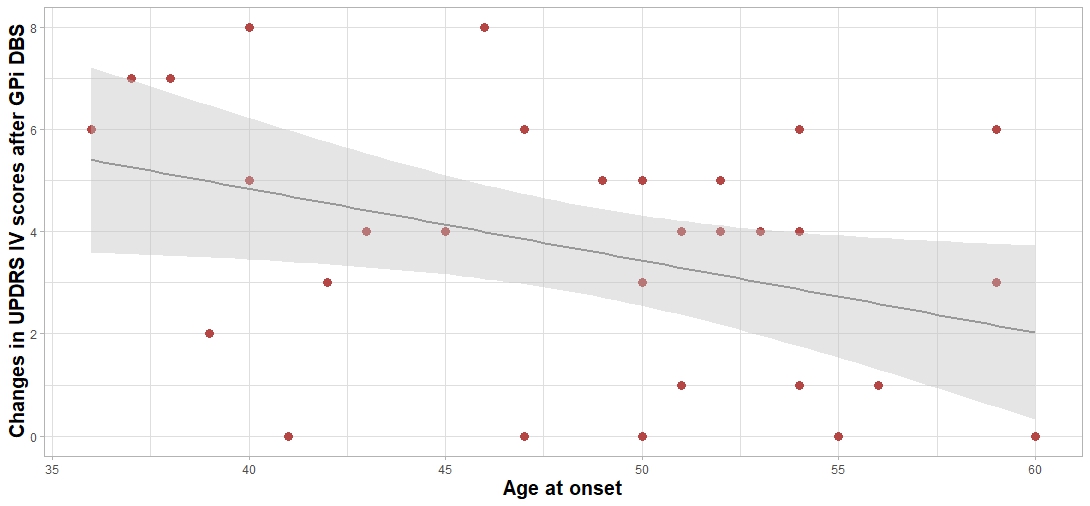


**Supplementary Figure 3.** The correlation between age at onset of PD and changes in UPDRS IV score in the after GPi DBS in patients with advanced PD. There is a negative linear relationship between two variables. Pearson’s r = -0.39, p = 0.032. The correlation is significant at a 0.05 level. PD = Parkinson’s disease, GPi = Globus pallidus interna, DBS = Deep brain stimulation, UPDRS = Unified Parkinson’s Disease Rating Scale.

|  | **Age at onset (yr)** | **Disease duration (yr)** | **Motor**  **phenotype** | **Levodopa responsiveness (%)** | **Baseline LEDD (mg)** | **DBS motor**  **responsiveness (%)** | **mean voltage (V)** | **mean**  **pulse width (μs)** | **mean frequency (Hz)** | **Post DBS LEDD (mg)** |
| --- | --- | --- | --- | --- | --- | --- | --- | --- | --- | --- |
| P1 | 54 | 14 | PIGD | 84.2 | 1614 | 42.1 | 2.2 | 75 | 90 | 1614 |
| P2 | 40 | 14 | TD | 87.2 | 1258 | 87.2 | 2.85 | 75 | 135 | 1258 |
| P3 | 46 | 10 | PIGD | 53.8 | 666 | 38.5 | 2.65 | 60 | 127.5 | 1000 |
| P4 | 55 | 14 | PIGD | 76.7 | 1091 | 13.3 | 2.55 | 75 | 115 | 1656 |
| P5 | 51 | 15 | PIGD | 58.1 | 1173 | -19.4 | 3.55 | 75 | 130 | 1443 |
| P6 | 53 | 11 | PIGD | 85.7 | 1399 | 46.4 | 3.35 | 60 | 130 | 1299 |
| P7 | 38 | 18 | PIGD | 34.6 | 960 | 64.7 | 3.5 | 60 | 155 | 1160 |
| P8 | 60 | 8 | PIGD | 70.9 | 1048 | -29.0 | 3.2 | 60 | 140 | 1164 |
| P9 | 40 | 13 | PIGD | 60.9 | 1025 | 32.6 | 2.9 | 60 | 130 | 998 |
| P10 | 54 | 11 | PIGD | 74 | 1514 | 32 | 2.85 | 60 | 130 | 1049 |
| P11 | 56 | 12 | PIGD | 65.1 | 1148 | 26.7 | 3.15 | 60 | 130 | 1075 |
| P12 | 43 | 11 | PIGD | 74.7 | 1730 | -5.7 | 2.7 | 60 | 130 | 1500 |
| P13 | 41 | 17 | PIGD | 49.4 | 1624 | 37.3 | 3.55 | 60 | 130 | 2164 |
| P14 | 50 | 14 | PIGD | 56.7 | 759 | -10.0 | 3.1 | 60 | 130 | 759 |
| P15 | 52 | 15 | PIGD | 64.9 | 1238 | 36.5 | 2.85 | 60 | 130 | 1038 |
| P16 | 59 | 10 | PIGD | 69.6 | 948 | 10.1 | 3.05 | 60 | 115 | 1480 |
| P17 | 45 | 23 | PIGD | 70.8 | 1774 | 25.8 | 2.0 | 60 | 130 | 1774 |
| P18 | 36 | 12 | PIGD | 68.7 | 1248 | 59.4 | 2.4 | 60 | 130 | 1115 |
| P19 | 47 | 22 | PIGD | 39.1 | 998 | 31.1 | 2.3 | 60 | 100 | 865 |
| P20 | 42 | 14 | PIGD | 42.3 | 975 | 38.5 | 3.15 | 60 | 130 | 1063 |
| P21 | 47 | 11 | PIGD | 76.4 | 958 | 0.0 | 2.1 | 60 | 130 | 825 |
| P22 | 51 | 9 | PIGD | 96.7 | 1374 | 24.6 | 2.85 | 60 | 115 | 1308 |
| P23 | 50 | 7 | PIGD | 56.6 | 948 | 16.3 | 3.8 | 60 | 130 | 1210 |
| P24 | 52 | 17 | PIGD | 42.8 | 1173 | 30.4 | 3.5 | 60 | 100 | 998 |
| P25 | 54 | 13 | TD | 72.3 | 1050 | 36.6 | 2.75 | 60 | 130 | 863 |
| P26 | 39 | 20 | PIGD | 37.1 | 1207 | 40.0 | 2 | 60 | 130 | 1207 |
| P27 | 37 | 24 | PIGD | 83.3 | 1275 | 42.4 | 2.7 | 60 | 145 | 875 |
| P28 | 54 | 17 | PIGD | 64.9 | 1350 | 34.0 | 2.8 | 60 | 130 | 1100 |
| P29 | 50 | 11 | PIGD | 62.8 | 1150 | -13.4 | 2.9 | 90 | 130 | 1150 |
| P30 | 59 | 9 | PIGD | 69.1 | 1400 | 11.1 | 3 | 90 | 100 | 1498 |
| P31 | 49 | 16 | IND | 58.7 | 1113 | 47.7 | 2.4 | 60 | 130 | 525 |

**Supplementary table 1.** Clinical features, motor phenotypes and DBS programming parameters at 12 months after surgery in patients with PD who underwent GPi DBS. The TD (a ratio of > 1.5) and PIGD (a ratio of < 1.0) subtypes were classified according to the ratio of mean tremor score from UPDRS II and III (items 16,20, and 21) to the mean PIGD score (items 13-15, 29 and 30). DBS, Deep brain stimulation; GPi, Globus pallidus interna; TD, Tremor-dominant; PIGD, postural instability and gait difficulty; IND, Indeterminate; PD, Parkinson’s disease.

|  | **Univariate analysis** | | | **Multivariate analysis** | | |
| --- | --- | --- | --- | --- | --- | --- |
|  | **Coefficient** | **95% CI** | **p** | **Coefficient** | **95% CI** | **p** |
| Sex (male) | -5.606 | (-13.54, 2.33) | 0.159 | -7.341 | (-15.17, 0.49) | 0.065 |
| Age at onset | 0.555 | (-0.01, 1.12) | 0.056 | 0.666 | (0.08, 1.26) | 0.029 |
| Disease duration | -0.125 | (-1.10, 0.85) | 0.794 | -0.383 | (-1.56, 0.80) | 0.508 |
| MMSE | -0.345 | (-1.63, 0.94) | 0.586 | 0.643 | (-0.60, 1.90) | 0.296 |
| Preoperative UPDRS III score | 0.352 | (0.07, 0.64) | 0.017 | 0.508 | (0.18, 0.83) | 0.004 |
| Levodopa responsiveness (%) | -0.141 | (-0.40, 0.12) | 0.283 | -0.084 | (-0.41, -0.24) | 0.595 |
| LEDD (mg) | -0.002 | (-0.02, 0.01) | 0.846 | 0.004 | (-0.01, 0.00) | 0.586 |
| Striatal SBR value | 0.514 | (-9.85, 10.88) | 0.920 | -1.782 | (-13.38, 9.81) | 0.753 |

**Supplementary Table 2.** Univariable and multivariate regression analysis of the factors associated with the UPDRS III scores in the medication off stimulation on state 12 months after GPi DBS in patients with advanced Parkinson' disease. DBS, deep brain stimulation; GPi, globus pallidus interna; MMSE, Mini-Mental State Examination; SBR, specific binding ratio; LEDD, levodopa equivalent daily dose; UPDRS, Unified Parkinson’s Disease Rating Scale.

|  | **Univariate analysis** | | | **Multivariate analysis** | | |
| --- | --- | --- | --- | --- | --- | --- |
|  | **Coefficient** | **95% CI** | **p** | **Coefficient** | **95% CI** | **p** |
| Sex (male) | 0.617 | (-0.63, 1.86) | 0.318 | 0.201 | (-1.21, 1.61) | 0.770 |
| Age at onset | -0.083 | (-0.17, 0.01) | 0.063 | -0.136 | (-0.24, -0.03) | 0.014 |
| Disease duration | -0.06 | (-0.21, 0.09) | 0.417 | -0.167 | (-0.38, 0.05) | 0.117 |
| MMSE | 0.078 | (-0.12, 0.27) | 0.422 | 0.039 | (-0.19, 0.26) | 0.721 |
| Preoperative UPDRS III | 0.001 | (-0.05, 0.05) | 0.982 | 0.023 | (-0.04, 0.08) | 0.428 |
| Levodopa responsiveness (%) | 0.015 | (-0.04, 0.09) | 0.46 | 0.035 | (-0.02, 0.09) | 0.217 |
| LEDD (mg) | -0.001 | (-0.03, 0.06) | 0.608 | -0.001 | (0.00, 0.00) | 0.430 |
| Striatal SBR values | 0.294 | (-1.130, 1.88) | 0.708 | -0.764 | (-2.85, 1.32) | 0.455 |

**Supplementary table 3.** Univariable and multivariate regression analysis of the factors associated with the changes in dyskinesia scores 12 months after GPi DBS in patients with advanced Parkinson' disease. DBS, deep brain stimulation; GPi, globus pallidus interna; MMSE, Mini-Mental State Examination; SBR, specific binding ratio; LEDD, levodopa equivalent daily dose; UPDRS, Unified Parkinson’s Disease Rating Scale.

|  | **Univariate analysis** | | | **Multivariate analysis** | | |
| --- | --- | --- | --- | --- | --- | --- |
|  | **Coefficient** | **95% CI** | **p** | **Coefficient** | **95% CI** | **p** |
| Sex (male) | 0.775 | (-2.78, 1.83) | 0.143 | 0.909 | (-0.46, 2.28) | 0.181 |
| Age at onset | -0.016 | (-0.10, 0.06) | 0.684 | 0.001 | (-0.10, 0.10) | 0.989 |
| Disease duration | 0.045 | (-0.08, 0.06) | 0.476 | -0.017 | (0.22, 0.19) | 0.865 |
| MMSE | -0.013 | (-0.18, 0.16) | 0.881 | -0.019 | (-0.24, 0.20) | 0.861 |
| Preoperative UPDRS III | 0.011 | (-0.03, 0.05) | 0.586 | 0.002 | (-0.05, 0.06) | 0.931 |
| Levodopa responsiveness (%) | -0.005 | (-0.04, 0.03) | 0.775 | -0.007 | (-0.06, 0.05) | 0.794 |
| LEDD (mg) | 0.001 | (-0.00, 0.00) | 0.641 | 0.001 | (-0.06, 0.05) | 0.707 |
| Striatal SBR values | -0.747 | (-2.10, 060) | 0.266 | -0.841 | (-2.87, 1.19) | 0.399 |

**Supplementary table 4.** Univariable and multivariate regression analysis of the factors associated with the changes in motor fluctuation scores 12 months after GPi DBS in patients with advanced Parkinson' disease. DBS, deep brain stimulation; GPi, globus pallidus interna; MMSE, Mini-Mental State Examination; SBR, specific binding ratio; LEDD, levodopa equivalent daily dose; UPDRS, Unified Parkinson’s Disease Rating Scale.

|  | **Young-onset PD**  **(n =14)** | **older-onset PD**  **(n = 17)** | **p** | **adjusted p** |
| --- | --- | --- | --- | --- |
| **Baseline** |  |  |  |  |
| Women (%) | 8 (57) | 9 (53) | 0.870 | 0.984 |
| Age at onset (years) | 42.14 ± 4.11 | 53.76 ± 3.21 | < 0.001 | < 0.001 |
| Disease duration (years) | 16.07 ± 4.71 | 12.18 ± 3.03 | 0.009 | 0.069 |
| Age at surgery (years) | 58.21 ± 5.87 | 65.94 ± 3.72 | < 0.001 | 0.012 |
| Mini-Mental State Examination (MMSE) | 26.29 ± 4.08 | 26.88 ± 2.45 | 0.953 | 0.984 |
| Hoehn & Yahr stage (medication off) | 3.21 ± 0.67 | 3.18 ± 0.39 | 0.984 | 0.984 |
| Levodopa responsiveness (%) | 59.79 ± 17.61 | 68.89 ± 12.68 | 0.105 | 0.294 |
| UPDRS III (medication off) | 47.43 ± 16.07 | 42.91 ± 10.70 | 0.357 | 0.578 |
| UPDRS III (medication on) | 19.82 ± 13.79 | 13.67 ± 7.44 | 0.377 | 0.578 |
| UPDRS IV | 9.07 ± 2.76 | 8.18 ± 2.01 | 0.305 | 0.540 |
| Dyskinesia | 3.50 ± 2.28 | 2.35 ± 1.58 | 0.109 | 0.294 |
| Motor fluctuation | 4.43 ± 1.02 | 4.71 ± 1.10 | 0.468 | 0.673 |
| Striatal SBR value | 0.68 ± 0.35 | 0.94 ± 0.42 | 0.080 | 0.294 |
| LEDD (mg) | 1200.79 ± 320.15 | 1198.65 ± 222.12 | 0.983 | 0.984 |
| **12 months after GPi DBS** |  |  |  |  |
| UPDRS III (medication off stimulation on) | 28.25 ± 12.29 | 34.53 ± 9.21 | 0.115 | 0.294 |
| UPDRS III (medication off stimulation off) | 35.35 ± 12.41 | 43.94 ± 10.57 | 0.046 | 0.212 |
| UPDRS III (medication on stimulation on) | 15.45 ± 11.86 | 19.09 ± 8.86 | 0.138 | 0.317 |
| UPDRS III (medication on stimulation off) | 20.79 ± 11.25 | 25.74 ± 11.78 | 0.173 | 0.355 |
| DBS responsiveness (%) | 38.54 ± 23.69 | 16.97 ± 22.77 | 0.015 | 0.086 |
| UPDRS IV | 4.43 ± 2.03 | 5.35 ± 1.77 | 0.185 | 0.355 |
| Dyskinesia | 1.29 ± 1.14 | 1.35 ± 1.37 | 0.984 | 0.984 |
| Motor fluctuation | 2.43 ± 1.55 | 2.71 ± 1.26 | 0.588 | 0.796 |
| LEDD (mg) | 1166.36 ± 416.84 | 1217.88 ± .256.06 | 0.676 | 0.864 |

**Supplementary table 5.** Comparison between young-onset PD patients and older-onset PD patients who underwent GPi DBS. DBS, Deep brain stimulation; GPi, Globus pallidus interna; LEDD, Levodopa equivalent daily dose; MMSE, Mini-Mental State Examination; PD, Parkinson’s disease; SBR, Specific binding ratio; UPDRS, Unified Parkinson’s Disease Rating Scale.
